# The Role of Next-Generation Probiotics in Obesity and Obesity-Associated Disorders: Current Knowledge and Future Perspectives

**DOI:** 10.3390/ijms24076755

**Published:** 2023-04-04

**Authors:** Natalia G. Vallianou, Dimitris Kounatidis, Dimitrios Tsilingiris, Fotis Panagopoulos, Gerasimos Socrates Christodoulatos, Angelos Evangelopoulos, Irene Karampela, Maria Dalamaga

**Affiliations:** 1 Department of Internal Medicine, Evangelismos General Hospital, 45-47 Ipsilantou Street, 10676 Athens, Greece; 2 First Department of Internal Medicine, University Hospital of Alexandroupolis, Democritus University of Thrace, 68100 Alexandroupolis, Greece; 3 Department of Biological Chemistry, Medical School, National and Kapodistrian University of Athens, 75 Mikras Asias Street, 11527 Athens, Greece; 4 Department of Microbiology, Sismanogleio General Hospital, 1 Sismanogleiou Street, 15126 Athens, Greece; 5 Roche Hellas Diagnostics S.A., 18-20 Amarousiou-Chalandriou Street, 15125 Athens, Greece; 6 2nd Department of Critical Care, Medical School, University of Athens, Attikon General University Hospital, 1 Rimini Street, 12462 Athens, Greece

**Keywords:** diabetes mellitus, microbiome, microbiota, obesity, probiotics

## Abstract

Obesity and obesity-associated disorders pose a major public health issue worldwide. Apart from conventional weight loss drugs, next-generation probiotics (NGPs) seem to be very promising as potential preventive and therapeutic agents against obesity. Candidate NGPs such as *Akkermansia muciniphila*, *Faecalibacterium prausnitzii*, *Anaerobutyricum hallii, Bacteroides uniformis, Bacteroides coprocola, Parabacteroides distasonis, Parabacteroides goldsteinii, Hafnia alvei, Odoribacter laneus* and *Christensenella minuta* have shown promise in preclinical models of obesity and obesity-associated disorders. Proposed mechanisms include the modulation of gut flora and amelioration of intestinal dysbiosis, improvement of intestinal barrier function, reduction in chronic low-grade inflammation and modulation of gut peptide secretion. *Akkermansia muciniphila* and *Hafnia alvei* have already been administered in overweight/obese patients with encouraging results. However, safety issues and strict regulations should be constantly implemented and updated. In this review, we aim to explore (1) current knowledge regarding NGPs; (2) their utility in obesity and obesity-associated disorders; (3) their safety profile; and (4) their therapeutic potential in individuals with overweight/obesity. More large-scale, multicentric and longitudinal studies are mandatory to explore their preventive and therapeutic potential against obesity and its related disorders.

## 1. Introduction

Obesity has become a major global public threat that is associated with a variety of disorders, including metabolic syndrome, type 2 diabetes mellitus, cardiovascular disease, nonalcoholic fatty liver disease, polycystic ovary syndrome, cancer, autoimmune disorders, and the severity of COVID-19 [1,2,3,4,5,6,7,8,9,10,11,12,13,14,15]. Based on recent WHO data, the worldwide prevalence of overweight and obesity in the adult population is greater than 39% and 13%, respectively [16].

As the obesity epidemic is on the rise worldwide, novel treatment strategies are being developed [12,17]. Apart from classical treatment options, such as glucagon-like peptide-1 (GLP-1) receptor agonists [18,19,20], researchers have also focused on the microbiome as a potential target to combat obesity [12,20,21,22,23].

The term microbiome refers to the sum of genes from the total amount of bacteria, viruses, fungi, archaea and protozoa inhabiting the human body. Amongst them, most of the microorganisms in the human body reside in the gut, comprising the human gut microbiota [21,22,23,24]. There, trillions of microorganisms interact with each other and produce a variety of metabolites, which play a crucial physiological role [25,26,27]. More specifically, microorganisms in the gut produce short-chain fatty acids (SCFAs), such as butyric acid, propionic acid and acetate, which account for several beneficial functions. SCFAs have been implicated in strengthening the integrity of the gut barrier as well as in reducing serum endotoxin levels and serum inflammatory markers [27,28,29]. Nowadays, the notion that the exogenous administration of these beneficial microbes can improve human health is widely accepted. In 1974, the term “probiotics” was introduced to describe “live microorganisms that confer a health benefit when consumed in adequate amounts”. This was the final definition proposed by the World Health Organization (WHO) in 2002 [30]. The most famous probiotics are *Lactobacillus* strains and *Bifidobacteria,* which have been used in dietary supplements and functional foods [31]. *Saccharomyces boulardii* is another well-known probiotic that has been administered for the prophylaxis of antibiotic-associated diarrhea [32]. *Lactobacillus* and *Bifidobacterium* strains have been reported to ameliorate obesity and obesity-associated disorders in rodents [33,34,35,36,37,38,39,40,41,42,43,44]. In addition, *Saccharomyces boulardii* has also been demonstrated to reduce fat mass and systemic inflammation in rodents with obesity and diabetes [45]. However, with the recent advent of multi-omics and other technological developments, new species with beneficial health effects have been identified, called next-generation probiotics (NGPs). Currently, there has been a tendency to identify and test these NGPs in the context of specific diseases. NGPs are currently in the spotlight of research, and there is a growing body of evidence with regard to their therapeutic potential in obesity and its related disorders. In this review, we aim to explore (1) current knowledge regarding NGPs; (2) their utility in obesity and obesity-associated disorders; (3) their safety profile; and (4) their therapeutic potential in individuals with overweight/obesity.

## 2. A Brief Synopsis of Classical Probiotics and Their Effects on Obesity and Associated Disorders

The available preclinical and clinical evidence points toward the presence of the beneficial effects of certain classical probiotics on obesity and related disorders. Reported results have been variable and partially strain-specific, with heterogeneity amongst studies being an additional factor contributing to equivocal results. The proposed mechanisms of action for classical probiotics in obesity include the modulation of gut flora and amelioration of intestinal dysbiosis, improved intestinal barrier function with a reduction in endotoxemia, low-grade inflammation and oxidative stress, which, in turn, beneficially affect aspects of energy metabolism in adipose and other target tissues [46,47,48,49].

The administration of different strains of *Lactobacillus rhamnosus*, *plantarum, curvatus or gasseri* in diet-induced obese (DIO) mice has been associated with a multitude of benefits regarding adiposity and its related complications, such as a reduction in weight and visceral fat as well as a decrease in glucose, insulin and triglyceride levels. These probiotics have also been associated with a reduction in insulin resistance (IR) and proinflammatory cytokines, as well as a concomitant increase in interleukin-10 (IL-10) and an improvement in fatty liver indices [43,50,51,52,53,54,55]. Regarding human studies, a 12-week supplementation of *L. gasseri* BNR17 in adults with overweight or grade I obesity resulted in significant reductions in visceral fat and weight circumference in comparison to a placebo without affecting circulating biochemical profiles [56]. In another randomized, placebo-controlled trial, a 24-week supplementation of the *L. rhamnosus* CGMCC1.3724 strain to individuals with obesity led to significant weight reductions among female participants [33]. On the other hand, the integration of an *L. rhamnosus* probiotic supplement on a 20-week, low-glycemic index, hypocaloric dietary intervention in women with overweight or obesity and polycyctic ovary syndrome had no additive effects on anthropometry and circulating lipid profiles after 20 weeks [57]. Additionally, a recent meta-analysis of nine clinical trials on *Lactobacillus* supplementation in individuals who are overweight and obese has shown beneficial effects on total and low-density lipoprotein (LDL) cholesterol, fasting glucose and triglyceride concentrations [58].

The supplementation of different *Bifidobacterium* species has been studied in rodent obesity models with strain-dependent effects. In DIO mice, *Bifidobacteria* FS31-12, L66-5, M13-4, and L75-4 reduced serum triglycerides and liver fat content, although only L66-5 showed weight-loss properties [59]. Furthermore, *B. adolescentis* supplementation has shown weight-lowering effects and an improvement in steatosis and steatohepatitis in mouse and rat models [60,61]. The treatment of rodents with other *Bifidobacterium* strains has resulted in the reduction in visceral fat and IR, as well as glucose-lowering and diabetes-preventing effects [62,63,64]. In human trials, the supplementation of *Bifidobacteria*-containing probiotic formulations (*B. bifidum; B. breve, B. longum, B. animalis* subsp. *lactis*) has been associated with improvements in adiposity, glycemia, insulin resistance and fatty liver indices, with potential species-specific effects [65].

The administration of *Saccharomyces boulardii* to *db/db* mice with diabetes for 4 weeks resulted in reductions in the body weight and fat mass, improvements in fatty liver, and lowering of inflammatory indices. These effects were likely mediated through the modulation of gut microbiota [45]. Furthermore, *S. boulardii* reduced oxidative stress and liver injury in mice with streptozotocin-induced diabetes mellitus [66]. In a placebo-controlled randomized trial, the co-administration of *S. boulardii* and superoxide dismutase for 60 days among patients with obesity resulted in a reduced fat mass with the preservation of lean mass and improvements in IR [67].

## 3. Next-Generation Probiotics: The Contribution of Technology

The majority of microorganisms inhabiting the gut have remained unidentified until recently, as they are mostly anaerobic bacteria, which are difficult to cultivate [68]. However, modern microbiology has revolutionized our ability to identify previously unidentified microbes of the gut microbiota. In particular, with the advent of the polymerase chain reaction (PCR) of the 16 S rRNA gene, as well as next-generation sequencing (NGS) and the use of bioinformatics, we are now able to accurately identify various bacterial strains. Thus, PCR and NGS, with the use of various platforms, such as Illumina, have made possible the detection of the bacteria residing in the gut [69]. In this way, we can characterize the microorganisms associated with several disease states, including obesity and obesity-related disorders. In addition, we can identify species that are less abundant in obesity and its associated disorders, which may be associated with beneficial effects regarding obesity. Among these, *Akkermansia muciniphila, Faecalibacterium prausnitzii, Eubacterium hallii* (recently reclassified into *Anaerobutyricum hallii* and *Anaerobutyricum soenhgenii* [70], *Bacteroides uniformis, Bacteroides coprocola, Parabacteroides distasonis, Parabacteroides goldsteinii, Hafnia alvei, Odoribacter laneus* and *Christensenella minuta* have been proposed as NGPs, which could prove especially useful in the treatment of obesity and obesity-associated disorders [71]. Table 1 depicts major studies investigating the administration of various NGPs and their effects on overweight/obese patients. Notably, apart from culturomics, the multi-omics approach, i.e., the identification of genes (genomics), their phenotypes (epigenomics), mRNA (transcriptomics), proteins (proteomics) and various metabolites (metabolomics), can improve our understanding of the composition of the gut microbiota [71]. Collectively, high-throughput technology has shed light on the interplay between bacteria, viruses, fungi, archaea and protozoa comprising the gut microbiota.

## 4. Preventive and Therapeutic Potential of NGPs in Obesity and Obesity-Related Disorders

### 4.1. Akkermansia muciniphila

Among NGPs, *Akkermansia muciniphila (A. muciniphila)* has been the best-studied and perhaps the most promising NGP until now. *A. muciniphila* is a Gram-negative, anaerobic and non-motile, oval-shaped bacterium which belongs to the phylum *Verrucomicrobia* [72]. It colonizes the gut very early, during the first year of life, via human milk [73]. Thereby, it is highly abundant in the gut of infants and healthy adults, accounting for 0.5–5% of the human gut microbiota [72,73,74]. Its beneficial effects are mainly attributed to its ability to degrade mucin: a major component of mucus in the gut [74]. The degradation of mucin leads to a counterbalanced increased production of mucin in the gut, which results in the restoration of intestinal barrier integrity [75,76]. *A. muciniphila* uses mucin as a carbon and nitrogen source, as well as an energy source. It improves intestinal barrier integrity by the increased expression of tight junction proteins such as claudin-3, -4, -15 and occludin [75,76,77]. Furthermore, it reduces the production of bacterial lipopolysaccharide (LPS) in the gut, exerting anti-inflammatory effects [78,79]. Notably, its administration to overweight/obese individuals has been shown to decrease serum LPS levels as well as white blood cell count (WBC) [78,79]. These reductions have been associated with the anti-inflammatory properties of *A. muciniphila* [78,79].

Being an anaerobe bacterium, A. muciniphila requires specific conditions in order to be cultivated. Apart from the anaerobic or microaerophilic environment, another requirement is the addition of mucin from animal sources. However, since the first isolation of *A. muciniphila* in 2004, novel synthetic media have been applied to facilitate its cultivation. These synthetic media do not preclude their administration to human beings, as safety is always an absolute priority for NGPs [72,80,81]. In a comparison between this synthetic medium and an animal mucin-based counterpart, it was documented that *A. muciniphila* was cultivated in both media and was equally effective in mice. In particular, Plovier et al. demonstrated that its administration in rodents was associated with less weight gain and improved glucose tolerance [80]. In addition, they showed that, contrary to the complete inactivation of *A. muciniphila* by autoclaving, pasteurization resulted in the sustained and surprisingly augmented beneficial effects of this NGP. More specifically, rodents fed with a high lipid diet, which received a pasteurized form, exhibited the same weight gain as mice who were fed with a controlled diet. This augmentation was attributed to a decrease in the energy absorption caused by this NGP. In addition, the pasteurized form improved glucose tolerance and hepatic insulin resistance; it also diminished diet-induced endotoxemia [80].

Apart from its use in animal models, *A. muciniphila* has been administered to overweight/obese participants. In a landmark study, Depommier et al. administered 10^10^ bacteria daily for 3 months in the alive or pasteurized form in 40 participants. Thirty-two overweight/obese participants completed the study. The administration of *A. muciniphila* for 3 months was safe and well tolerated. The pasteurized form of *A. muciniphila* improved insulin sensitivity, insulinemia by approximately 30%, and total cholesterol levels while decreasing body weight and fat mass as well [79]. Moreover, decreases in serum gamma-glutamyl-transferase (γ-GT) and serum aspartate-aminotransferase (AST) levels were noted in the group that received *A. muciniphila* in its pasteurized form compared to a placebo. These improvements in hepatic indices, especially the 24% decrease in serum γ-GT levels, may indicate an amelioration in the deposition of fatty acids in the liver and, thus, the beneficial effect of non-alcoholic fatty liver disease (NAFLD) [79,80,81]. In addition, WBC, as well as serum LPS levels, were also statistically significantly reduced [79]. Regarding adverse effects, nausea and flatulence were rarely reported [79].

**Figure 1:**
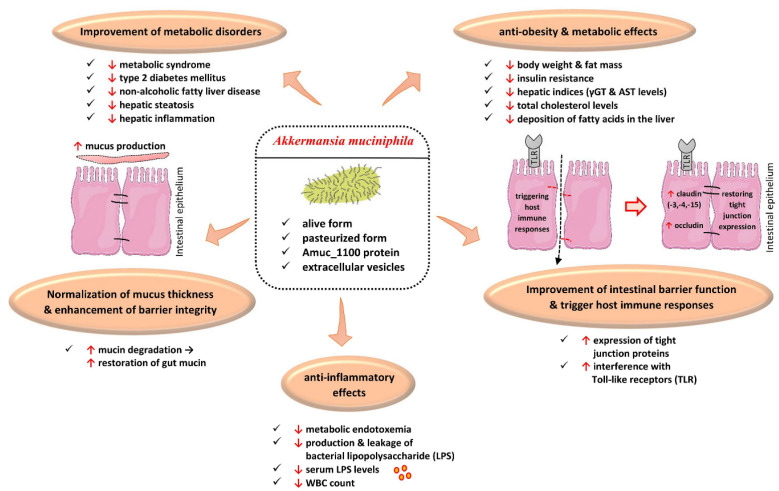
Effects of *Akkermansia muciniphila* and its derived products in obesity and its related disorders. ↑, augmentation; ↓, reduction; AST, aspartate-aminotransferase; γGT, Gamma-Glutamyl-Transferase; WBC, white blood cell. (All images originate from the free medical site http://smart.servier.com/ by Servier licensed under a Creative Commons Attribution 3.0 Unported License; accessed on 2 February 2023).

Overall, the administration of *A. muciniphila* as an NGP may have a promising future in the treatment of obesity and its related disorders [72,73,74,75,76,78,79,80,81,82] (Figure 1).

However, there are many issues still to be resolved. A pili-like protein on the outer membrane of *A. muciniphila* named Amuc_1100 was demonstrated to interfere with Toll-like receptors 2 and 4 (TLR-2 and TLR-4) [83,84,85,86], which are associated with host immune responses. With the use of proteomics, the Amuc_1100 protein has been shown to be among the most abundant proteins in the outer membrane of *A. muciniphila.* In addition, *A. muciniphila* extracellular vesicles can play a crucial role in host–bacteria interactions. For example, these extracellular vesicles have been implicated in a reduction in body weight as well as in the alleviation of inflammation and the amelioration of intestinal barrier integrity in obese animal models [87]. However, to date, it remains unknown whether these extracellular vesicles contain the Amuc_1100 protein or other proteins that exhibit the abovementioned potential [83,84,85,86].

These unanswered questions must be clarified before the wide therapeutic application of *A. muciniphila,* either alive, in its pasteurized form, or as extracellular vesicles for the treatment of obesity and its related disorders. As there is a growing interest in NGPs regarding obesity and obesity-associated disorders, future research will shed light on unresolved issues.

### 4.2. Faecalibacterium prausnitzii

*Faecalibacterium prausnitzii (F. prausnitzii)* is a Gram-positive, anaerobe, non-motile rod which accounts for 5–15% of the human gut microbiota composition [88,89,90]. Despite its relatively high abundance in healthy adults, it has been documented to decrease among patients with obesity, metabolic syndrome and NAFLD [90,91,92,93]. Its presence has been associated with anti-inflammatory properties as well as improved gut barrier integrity [21,25,93,94]. Indeed, *F. prausnitzii’s* administration in animal models has resulted in improvements in indices of NAFLD [95]. More specifically, among the 12 strains administered to HFD-mice (high-fat diet-fed mice) for 3 months, five specific strains led to an amelioration of various metabolic characteristics, such as glucose intolerance, hepatic steatosis, inflammation and oxidative stress in an animal model of NAFLD. Moreover, two strains, LC49 and LB8, increased the production of SCFAs and modified the composition of gut microbiota [95]. Nowadays, it has been widely accepted that the increased production of SCFAs, such as butyric acid, propionate and acetate, is implicated in the restoration of the imbalance of gut homeostasis. SCFAs are suggested to be amongst the major contributing factors of the homeostatic state (gut symbiosis) as opposed to the state of gut dysbiosis [21,25,94,95,96]. Collectively, *F. prausnitzii* has been associated with an enhancement in SCFAs, especially butyrate, and an amelioration of NAFLD markers. In addition, it has been documented to inhibit the activation of Nuclear Factor kappa B (NF-κB), thereby, exerting anti-inflammatory properties and diminishing chronic inflammation in the gut [97,98]. Apart from its anti-inflammatory potential, *F. prausnitzii* can promote the integrity of the intestinal barrier by increasing the tight junction proteins occludin and E-cadherin. In this way, it can mitigate the permeability of the gut barrier, enhancing the production of mucous [98,99].

### 4.3. Eubacterium hallii

*Eubacterium hallii (E. hallii)* is a Gram-positive, anaerobe bacterium, which is capable of producing butyrate from lactate and acetate in low pH conditions, as well as from monosaccharides, in the intestine [100,101]. As a butyrate producer, it has gained much attention lately as a potential NGP. Udayappan et al. administered *E. hallii* in rodents in a live and heat-killed bacterial form. The alive form of *E. hallii* was documented to increase the production of butyrate, improve the metabolism of bile acid and modulate the composition of the gut microbiota in diabetic mice [102]. Furthermore, the live form of *E. hallii* ameliorated insulin sensitivity while increasing energy expenditure in diabetic mice [102]. It has been demonstrated that glucose intolerance and hyperglycemia may increase the intestinal barrier’s permeability, leading to chronic gut inflammation [103,104]. Therefore, this improvement in insulin sensitivity and energy expenditure achieved by the administration of *E. hallii* may account for its potential beneficial effects [102,103,104]. Nevertheless, despite its promising features, there is a long way ahead before *E. hallii* can be used as an NGP.

### 4.4. Bacteroides uniformis and Bacteroides coprocola

*Bacteroides* are Gram negative, obligatory anaerobe rods, with a high abundance in the human gut microbiota, which are estimated to comprise the vast majority of its composition [105]. Yan et al. enrolled 41 individuals with a normal body mass index (BMI) divided into two groups: one with a low visceral fat area (L-VFA) and one with a high visceral fat area (H-VFA) according to computed tomography estimations. They further studied gut composition in the two groups and found significant differences. In particular, with the use of whole genome shotgun sequencing, they showed that *Bacteroides plebeius (B. plebeius)* and *Bacteroides uniformis (B. uniformis)* were highly abundant in those with L-VFA. Therefore, they concluded that *B. plebeius* and *B. uniformis* might serve as NGPs. Furthermore, *B. uniformis* was negatively correlated with serum LDL-cholesterol levels [106]. In general, *B. uniformis* has already been documented to possess beneficial properties against obesity and metabolic disorders [107]. *B. uniformis* has been related to weight loss, reduced serum cholesterol and triglycerides levels and an improvement in hepatic steatosis in animal models. In addition, it has been associated with decreased serum leptin levels, a fasting glucose concentration, and improved glucose tolerance [107]. *B. plebeius* has been suggested as a potential therapeutic target for patients with type 2 diabetes mellitus (T2DM) based on its considerably higher abundance in patients with obesity and T2DM compared with the controls [108]. *Bacteroides coprocola (B. coprocola)* has been negatively associated with liver stiffness among male patients with metabolic dysfunction-associated fatty liver disease (MAFLD) [108]. In particular, among 85 individual males with MAFLD, patients with increased liver stiffness measurements (LSM), as defined by LSM > 7.4 kPa, had a decreased abundance of *B. coprocola* in the gut using whole genome sequencing [109]. Furthermore, Cuffaro et al. showed that *B. coprocola* AS101, *B. uniformis* PF-BaE8 and PF-BaE13 strains exhibited combined anti-inflammatory properties as well as an improvement in the intestinal barrier function [71]. Therefore, these specific strains might be NGP candidates for future research in obesity and its associated disorders.

### 4.5. Parabacteroides distasonis and Parabacteroides goldsteinii

*Parabacteroides* are Gram-negative, obligatory anaerobe, non-motile rods that colonize the human intestine [110]. *Parabacteroides distasonis (P. distansonis)* may be another promising NGP candidate due to its ability to reduce weight gain, hyperglycemia and hepatic steatosis in obese and HFD-mice [111]. More specifically, the oral administration of *P. distasonis* CGMCC 1.30169 reduced liver cholesterol, free fatty acids and triglyceride concentrations while also enhancing the production of succinate and secondary bile acids in the gut. In this animal model, all the beneficial properties resulted from the administration of living rather than heat-killed *P. distasonis* [111]. In addition, *P. distasonis* AS93 was demonstrated to exhibit anti-inflammatory properties as well as protective effects on the gut barrier function. Moreover, the same study reported that it also promoted the secretion of GLP-1 [71]. In addition, *P. distasonis* was suggested to exert its anti-inflammatory effects through the inhibition of NF-κB and the PI3K/Akt/mTOR pathways [112,113]. *P. goldsteinii* has been shown to correlate with HDL-cholesterol and IL-10, whereas it was inversely related to serum glucose and triglyceride levels in HFD-mice [114,115]. In addition, it has been related to less weight gain and reductions in inflammatory indices, such as the Tumor Necrosis Factor (TNF)-a and interleukin (IL)-1β [114,115]. Moreover, the oral administration of a live form of *P. goldsteinii* ATCC BAA-1180 in HFD-mice has been associated with reduced weight gain and the amelioration of various metabolic parameters, such as inflammation and insulin resistance [114,115]. Overall, despite the fact that both *P. distasonis* and *P. goldsteinii* have just started to be in the spotlight of microbiome research, they have shown promising effects regarding obesity and its related disorders.

### 4.6. Hafnia alvei

*Hafnia alvei (H. alvei)* is a Gram-negative, facultative anaerobic rod. The genus Hafnia is one of 40 members of the Enterobacterales order [116,117]. Lucas et al. reported that the oral administration of the *H. alvei* HA4597^TM^ strain in HFD-mice resulted in reduced weight and fat mass gain and decreased food intake [116]. In addition, the group that was administered with *H. alvei* HA4597^TM^ showed decreases in serum glucose, triglycerides and anino-transferase levels [116]. Legrand et al. found that oral gavage with *H. alvei* in *ob/ob* and HFD-mice for 18 and 46 days, respectively, decreased the fat mass in both mice and reduced food intake in hyperphagic *ob/ob* mice. Overall, the administration of *H. alvei* HA4597^TM^ was well tolerated and had weight-lowering effects [117]. Based on the abovementioned findings, Dechelotte et al. embarked on the study of 236 overweight patients who were administered *H. alvei* HA4597^®®^ in conjunction with a moderately hypocaloric diet for 12 weeks. They observed a significant weight loss of at least 3% and even 4% of the baseline body weight [118]. This constitutes an important finding, considering that a weight loss of 3% to 5% of body weight is recommended by international guidelines for overweight patients [119]. This has also been related to reductions in the prevalence of T2DM and cardiovascular risk [119]. In addition, Dechelotte et al. documented a decrease in hip circumference and a subtle reduction in waist circumference and serum glucose levels [118]. The observed decrease in body weight may be attributed to metabolic properties in the eating behavior of the ClpB protein produced by *H. alvei* [118]. Notably, the development of this NGP has been based on the overproduction of the ClpB protein to enhance satiety pathways via the activation of the melanocortin receptor [117]. Indeed, the melanocortin network plays a crucial role in energy metabolism. This is mediated through the transmission of anorexigenic signals as well as an increase in the energy expenditure associated with peripheral lipolytic effects [118,119,120,121,122,123,124]. It is noteworthy that melanocortin receptors are also present in the intestinal mucosa, where they are readily accessible to gut bacteria-derived products, such as ClpB [118,119,120,121,122,123,124]. Other studies have also confirmed that the ClpB-like gene function in fecal microbiota is inversely associated with BMI and fat mass. In addition, obese patients have been documented to exhibit a low abundance of bacterial taxa expressing ClpB with the alpha-Melanocyte-stimulating hormone (MSH) homology. Overall, these findings support the fact that a high abundance of bacteria expressing the ClpB gene in the gut microbiota may be associated with increased satiety and decreased body weight [118,119,120,121,122,123,124]. Therefore, *H. alvei* could be a promising NGP candidate for obesity and its related disorders.

### 4.7. Odoribacter laneus

*Odoribacter laneus (O. laneus)* is a Gram-negative anaerobe rod that was isolated from the human feces of a healthy Japanese adult donor in 2010 [125]. Very recently, in 2022, Hueber-Ruano et al. characterized *O. laneus* as an NGP due to its ability to ameliorate insulin sensitivity and decrease inflammatory markers in a mouse model [126]. By administering *O. laneus* through oral gavage in obese mice, they documented a significant reduction in serum succinate levels. They concluded that excess succinate might be the result of a disrupted intestinal barrier integrity, especially in cases with gut dysbiosis. The augmentation of serum succinate levels could be attributed to the increased permeability of the gut barrier in the context of a leaking gut. Therefore, the administration of succinate-consuming bacteria, such as *O. laneus,* could be beneficial in T2DM and obesity. The increase in serum succinate levels has been associated with T2DM and obesity, characterized by chronic low-grade inflammation [127,128]. Additionally, succinate receptor 1 (SUCNR1) may play a pivotal role in the pathogenesis of T2DM and obesity. Indeed, it has been documented that the activation of SUCNR1 in macrophages could lead to the inflammation of adipose tissue and its infiltration with inflammatory cells [127,128]. Overall, *O. laneus* may present a promising potential based on its succinate-consuming properties.

### 4.8. Christensenella minuta

*Christensenella minuta* (*C. minuta*) is a Gram-negative, non-motile and non-spore-forming anaerobe rod belonging to the order *Clostridiales* of the family *Christensenellaceae* [129,130]. The family *Christensenellaceae* has been related to decreases in serum LDL-cholesterol, triglycerides, and alanine-aminotransferase (ALT) levels [131]. In addition, it is highly abundant in the gut of lean individuals [131,132]. Therefore, *C. minuta* has been proposed as an NGP. *C. minuta* DSM33407 has been very recently studied as a potential therapeutic agent in a diet-induced obesity animal model. Indeed, Mazier et al. confirmed that *C. minuta* DSM33407 decreased the obesogenic effects of diet in a mouse model while it improved glycemia and serum leptin levels, inhibited de novo lipogenesis and ameliorated the intestinal barrier integrity. Moreover, in a humanized simulator of the human intestinal microbial ecosystem model, they demonstrated that anti-obesity effects were also related to a decrease in the Furmicutes/Bacteroidetes ratio [130]. In a plethora of studies, the *Firmicutes/Bacteroidetes* ratio has been found to be increased in obesity. On the contrary, there is a decrease in the *Firmicutes/Bacteroidetes* ratio after weight loss in individuals with obesity [130,131,132,133].

### 4.9. Parabacteroides distasonis

*P. distansonis* is a Gram-negative, non-sporeforming, anaerbobic, rod-shaped bacterium that was originally isolated in the 1930s form clinical specimens and reclassified to the genus *Parabacteroides* in 2006 [134]. Wang et al. reported that treatment of ob/ob and DIO mice with *P. distansonis* resulted in weight loss and improvements in insulin sensitivity and hepatic steatosis. This was mediated through the production of succinate in the gut, which is bound to fructose-1,6-bisphosphatase, stimulating intestinal gluconeogenesis, as well as the activation of the Farnesoid X receptor pathway by secondary bile acids [111]. In humans, *P. distasonis* has been found to be more abundant in children with obesity compared to those with obesity and metabolic syndrome [135]. Haro et al. reported a significant increase in *P. distansonis* abundance in men with obesity after a 12-month adherence to a Mediterranean diet. It should be noted that no significant changes in anthropometric parameters were noted over the course of the study; however, there was an increase in the Insulin Sensitivity Index at the end of the intervention compared to the baseline, indicating improvements in whole-body insulin sensitivity [136]. Nevertheless, *S. distansonis* has not been universally associated with a beneficial metabolic profile; metformin treatment has been shown to decrease its abundance in high-fat-fed mice [137], while free access to a sugar-sweetened beverage in adolescent mice produced the opposite effect [138]. Furthermore, in contrast to the previously presented results, an increased abundance of *S. distansonis* was noted in patients with obesity and T2DM compared to lean control individuals in a case–control study by Yang et al. [139], while similar findings were reported in women with gestational diabetes mellitus by Kuang et al. [140].

## 5. Safety Profiles and Implementation of NGPs in the Prevention and Treatment of Obesity and Obesity-Related Disorders

NGPs are mostly anaerobic rods that are difficult to cultivate in practice. Additionally, there are other issues that need to be clarified. First, the exact dose of each NGP to exert a beneficial effect on humans needs to be determined in colony-forming units (CFU). In the study by Depommier et al., the dose was defined as 10^10^ CFU for *A. muciniphila* [78]. The exact dose of other NGPs in CFU also needs to be determined. Second, the optimal formulation of each NGP needs to be ascertained in order to increase the survival of live bacteria during storage and transit through the unfavorable conditions of the gastrointestinal microenvironment (low pH, peptic enzymes, bile acids, etc.) as well as their engraftment into the intestinal flora, when needed [141]. Third, more studies on the co-administration of two or more NGPs are required in order to determine synergistic actions which augment their beneficial potential. It is tempting to speculate that a combination of *H. alvei* HA4597^®®^, *A. muciniphila,* and other probiotics may provide beneficial additive or synergistic anti-obesity and metabolic effects in patients with obesity. The combination of two or more NGPs may maximize their beneficial effects; nevertheless, more studies are required to delineate any subsequent adverse effects. Probiotic-associated adverse effects, although scarce and typically of mild severity, are likely under-reported in human trials [142]. The available reports showing mild gastrointestinal disorders have been derived from the use of probiotics for indications other than obesity and dysmetabolism. Rarely, more severe complications due to invasive infections by formulated probiotic strains, such as bacteremia, overt sepsis or endocarditis, have been noted and typically observed in immunocompromised patients. Other speculative concerns include the lateral transmission of antibiotic-resistant genes from probiotic strains to gut bacteria and excessive immune stimulation in susceptible individuals, which may lead to autoimmune or autoinflammatory phenomena, although, to date, no such events have been reported [143]. Subsequently, safety regulations need to be strictly implemented and updated. For example, in the European Union, the regulations from the European Parliament and the Council of the European Union, regulation number 1924/2006, should be followed. In the United States, regulations from the Food and Drug Administration (FDA), with the characterization of “generally recognized as safe” (GRAS), should be implemented [69]. However, as new NGPs are currently emerging, the regulation of these should be constantly updated, while the presumed safety of NGPs should be harmonized with the needs as well as the safety of individuals who are overweight/obese [69,144].

## 6. Conclusions

As overweight/obesity poses a major public health problem worldwide, there is currently an urgent need for more preventive and therapeutic agents in our armamentarium against obesity and its related disorders. In this context, diet is the cornerstone of preventive and therapeutic interventions. A diet rich in fiber, such as vegetables and fruits, and low in animal proteins and fat should be adopted and followed. The Mediterranean diet and, lately, ketogenic diets are increasingly being recognized as important parameters in the fight against obesity [145,146,147]. Besides the use of agents with weight loss properties, such as GLP-1 analogs or tirzepatide [148,149], which possess agonist activities in both GLP-1 and glucose-dependent insulinotropic polypeptide (GIP) receptors, there is an emerging interest in the use of NGPs. The advent of sophisticated molecular methods has revolutionized the study of NGPs as potential preventive and therapeutic anti-obesity agents. Notably, there are numerous, mostly anaerobic bacteria that have been associated with weight loss. The study of NGPs has revealed a promising potential in the context of obesity, metabolic syndrome, NAFLD, and T2DM. However, there is much to be conducted in this research field. Currently available data have emerged from animal studies that may not present translational potential in humans [150]. Additionally, corresponding human trials are limited by their small sample sizes, monocentric design and short follow-up duration. The latter is particularly relevant regarding the assessment of adverse events, which are, in turn, likely to be under-reported [142]. Safety issues, as well as more eligible international regulations regarding their use, have to be clarified and implemented in the near future. Further large-scale, longitudinal studies with a multi-omics approach are mandatory in order to shed light on their therapeutic potential in obesity and obesity-associated disorders.

## Figures and Tables

**Table 1:** List of main studies associating the administration of NGPs and amelioration of metabolic dysfunction parameters.

Research NCT	Population	Study Groups	Center/P.I.	Title of the Study	Results
***List of Studies with Akkermansia muciniphila***					
NCT02637115	54 Participants, 18 to 70 y.o., Caucasian, BMI: 25–50.	I. Placebo II. Supplementation with live *Akkermansia muciniphila* 10^9^ CFU III. Supplementation with live *Akkermansia muciniphila* 10^10^ CFU IV. Supplementation with heat-killed *Akkermansia muciniphila*	Start date: 22 December 2015 Last posted: 17 May 2019. Completed Cliniques universitaires Saint-Luc Brussels, Belgium. P.I.: P. Cani.	✓ Evaluation of the Effects Associated with the Administration of *Akkermansia Muciniphila* on Parameters of Metabolic Syndrome.	✓ Pasteurized *A. muciniphila* ameliorated insulin sensitivity (+28.62 ± 7.02%, *p* = 0.002), ✓ ↓↓ insulinemia (−34.08 ± 7.12%, *p* = 0.006) ✓ ↓ serum total cholesterol (−8.68 ± 2.38%, *p* = 0.02), when compared to placebo. ✓ Pasteurized *A. muciniphila* ✓ ↓ BW (−2.27 ± 0.92 kg, *p* = 0.091) and fat mass (−1.37 ± 0.82 kg, *p* = 0.092) ✓ After 3 months of administering *A. muciniphila*, ↓↓ in biomarkers suggestive of liver dysfunction and inflammation. ✓ Supplementation was well tolerated.
NCT05114018	144 Participants 21 to 75 y.o. BMI: 25–40.	I. Placebo II. Supplementation with pasteurized *Akkermansia muciniphila*	Start date: 9 November 2021. Last posted: 15 September 2022. Clinical Research Center (CRC) Kiel GmbHKiel, Germany. Atlantia Food Clinical TrialsCork, Ireland. P.I.: P. Suenaert.	✓ Effect of Pasteurized *Akkermansia Muciniphila* on Insulin Resistance in Otherwise Healthy Subjects with Dysglycaemia.	✓ Still Recruiting
NCT05720299	120 Participants 18 to 65 y.o.BMI: 25–40.	I. Placebo II. Supplementation with *Akkermansia muciniphila*	Start date: 9 February 2023. The Seventh Affiliated Hospital of Southern Medical UniversityFoshan, Guangdong, China. P.I.: Yu Chen.	✓ A Clinical Study to Evaluate the Effects of *Akkermansia Muciniphila* on Insulin Resistance Among Obese Subjects.	✓ Still Recruiting
NCT04797442	60 Participants 18 to 60 y.o. BMI: 25–40.	I. Placebo II. WST01 Strain of live *Akkermansia muciniphila* Phase 2	Start date: 15 March 2021 Last posted: 25 October 2021. ✓ Dpt of Endocrinology of Shanghai Ruijin Hospital, Shanghai Jiaotong University School of Medicine Shanghai, Shanghai, China. P.I.: Weiking Wang.	✓ Effect of *Akkermansia Muciniphila* WST01 Strain in Overweight or Obese Patients with Type 2 Diabetes	✓ Still Recruiting
NCT05417360	108 Participants 20 to 70 y.o. BMI: 28–40.	I. Placebo II. Supplementation with pasteurized *Akkermansia muciniphila*	Start date: 22 June 2022 Last posted: 27 July 2022. Department of Human Biology, Maastricht University Medical Centre Maastricht, Limburg, Netherlands. P.I.: E. Blaak, E. Canfora.	✓ *Akkermansia* and Weight Maintenance (Amansia).	✓ Still Recruiting
***List of Studies with Hafnia alvei***					
NCT03657186	236 Participants 18 to 65 y.o. BMI: 25–29.9.	I. Placebo II. Supplementation with ProbioSatys^TM^	Start date: 4 September 2018 Last posted: 11 March 2020 Completed Analyze and Realize Berlin, Germany. P.I.: B. Grupe, J. Forstermann.	✓ Study to Evaluate Benefit of ProbioSatys™ on Weight Reduction in Overweight Subjects.	✓ In the *H. alvei* supplementation group, more subjects (+33%) achieved weight loss, when compared to a placebo. (54.9% vs. 41.4%, *p* = 0.048).The supplemented group reported a feeling of fullness (*p* = 0.009), while a significant reduction in hip circumference (*p* < 0.001) was noted, when compared to a placebo. ✓ ↓↓ Fasting glycemia at 12 weeks (*p* < 0.05) in the *H. alvei* supplementation group, when compared to a placebo. ✓ Supplementation was well tolerated.
NCT05170867	18 Participants 18 to 65 y.o. BMI: ≥ 35.	I. Placebo II. Supplementation with *Hafnia alvei* HA4597^TM^	Start date: 28 December 2021 Last posted: 4 November 2022. NOVA Medical School Faculdade de Ciências Médicas, Universidade NOVA de LisboaLisboa, Portugal. P.I.: C. Marques, C. Texeira.	✓ The Impact of *Hafnia Alvei* on Weight Loss and Glycaemic Control After Bariatric Surgery.	✓ Still Recruiting
***List of Studies with Eubacterium hallii***					
NCT04529473	100 Participants 21 to 69 y.o. BMI: 18.5–43.	I. Placebo II. Supplementation with live *Eubacterium hallii ≥* 10^9^ CFU	Start date: 27 August 2020 Last posted: 15 November 2022. Completed. Atlantia Food Clinical Trials, Chicago Chicago, Illinois, United States. Atlantia Food Clinical Trials Cork, Ireland. CPS Research Glasgow, Scotland, United Kingdom. P.I.: J. Ryan	✓ Efficacy and Safety of 12-weeks Supplementation of *Eubacterium Hallii* on Insulin Sensitivity and Glycaemic Control.	✓ Results have not been published yet.
***List of Studies with Christensenella minuta***					
NCT04663139	38 Participants 19 to 65 y.o. BMI: 18.5–24.9.	I. Placebo II. Supplementation with live Xla1 *Christensenella minuta* Phase 1	Start date: 10 December 2020 Last posted: 13 July 2021. Completed. Celerion Lincoln, Nebraska, United States. P.I.: Not reported.	✓ Xla1 *Christensenella Minuta*, Phase I, Randomized, Partially Placebo-controlled Double-blind Protocol, Evaluating Safety, Tolerability and Impact on the Gut Microbiota in Healthy Volunteers, Overweight and Obese Adults (CAUSALITY)	✓ Results have not been published yet.

**Abbreviations:** ↓ reduction; BW: Body Weight; BMI: Body Mass Index; P.I.: Primary Investigator; y.o.: years old.

## Data Availability

Not applicable.

## Abbreviations

ALT: Alanine-aminotransferase; AST: Aspartate-aminotransferase; BMI: Body Mass Index; CFU: Colony Forming Units; γGT: Gamma-Glutamyl-Transferase; FDA: Food and Drug Administration; GIP: Glucose-dependent Insulinotropic Polypeptide; GLP-1: Glucagon-like peptide-1; GRAS: Generally Recognized As Safe; HFD: High Fat Diet; H-VFA: High Visceral Fat Area; IL-10: Interleukin-10; IR: Insulin Resistance; LDL: Low-density lipoprotein; LSM: Liver Stiffness Measurement; LPS: Lipopolysaccharide; L-VFA: Low Visceral Fat Area; MAFLD: Metabolic dysfunction Associated Fatty Liver Disease; MSH: Melanocyte-stimulating hormone; NAFLD: Non Alcoholic Fatty Liver Disease; NF-κB: Nuclear Factor kappa B; NGPs: Next-Generation Probiotics; NGS: Next-Generation Sequencing; PCR: Polymerase Chain Reaction; SCFAs: Short Chain Fatty Acids; SUCNR1: Succinate receptor 1; T2DM: Type 2 Diabetes Mellitus; TNF-α: Tumor Necrosis Factor-alpha; WBC: white blood cell count; WHO: World Health Organization.
